# A Retrospective Analysis of Inhaled Nitric Oxide and Outcomes in Mechanically Ventilated Patients with COVID-19 ARDS

**DOI:** 10.3390/jcm15187302

**Published:** 2026-09-20

**Authors:** Abbie Evans, Shengping Yang, Noella Vinan-Vega, Jose Ramos, Pitchaporn Yingchoncharoen, Jerapas Thongpiya, Arunee Motes, Tarek Shihab, Cesar Peralta, Renee Garcia, Kenneth Nugent

**Affiliations:** 1 Division of Pulmonary and Critical Care, Department of Internal Medicine, Texas Tech University Health Science Center, 3601 4th St., Lubbock, TX 79430, USA; abbie.evans@ttuhsc.edu (A.E.);; 2 Pennington Biomedical Research Center, Baton Rouge, LA 70808, USA

**Keywords:** COVID-19, inhaled nitric oxide, acute respiratory distress syndrome, mechanical ventilation, pulmonary vasodilators, retrospective cohort

## Abstract

**Background:** Inhaled nitric oxide (iNO) is a selective pulmonary vasodilator used as rescue therapy in acute respiratory distress syndrome (ARDS) for transient improvement in oxygenation. Although multiple studies demonstrate improvements in oxygenation parameters, iNO has not consistently shown a mortality benefit or reduced ventilation duration. **Methods:** A retrospective cohort study was conducted of mechanically ventilated adults with SARS-CoV-2 (COVID-19) admitted to a medical intensive care unit who received iNO. Demographic characteristics, comorbidities, illness severity scores, timing of iNO initiation, and clinical outcomes were analyzed, with comparisons made between survivors and non-survivors. **Results:** A total of 94 patients were included. The mean age was 52.0 years (Standard Deviation (SD) 14.1), 70.2% were male, and the mean body mass index was 36.5 kg/m^2^ (SD 10.0). Hypertension (58.5%) and diabetes (36.2%) were the most common comorbidities. The mean Sequential Organ Failure Assessment (SOFA) score was 4.22 (SD 2.79), and the mean Acute Physiology and Chronic Health Evaluation II (APACHE II) score was 11.69 (SD 6.98). Overall, in-hospital mortality was 85.1% (80/94). The median time from intubation to iNO initiation was 7.6 days (mean 7.7 days). Survivors received iNO significantly earlier than non-survivors (mean 4.14 vs. 8.3 days after intubation, *p* = 0.024) and had a longer hospital length of stay (mean 37 vs. 23 days, *p* < 0.01). Duration of iNO therapy did not differ between groups (*p* = 0.12). **Conclusions:** In this cohort of critically ill patients with COVID-19 and severe respiratory failure, overall mortality was extremely high. Earlier initiation of inhaled nitric oxide was associated with improved survival, whereas duration of therapy was not. These findings suggest that the timing of iNO administration may be associated with clinical outcomes and warrant further prospective investigation to confirm this association.

## 1. Introduction

Inhaled nitric oxide (iNO) is a selective pulmonary vasodilator used in acute respiratory distress syndrome (ARDS) to transiently improve oxygenation. Its role in ARDS, including COVID-19-associated respiratory failure, remains controversial. Multiple studies have shown that iNO improves oxygenation parameters, with 45–66% of patients considered responders, defined as a ≥20% increase in PaO_2_/FiO_2_ [1,2,3,4,5,6,7]. These effects are most pronounced in patients with severe ARDS and may delay escalation to extracorporeal membrane oxygenation (ECMO) in select cases [5,7].

Despite improvements in oxygenation, iNO has not been shown to reduce mortality, the duration of mechanical ventilation, or intensive care unit (ICU) or hospital length of stay [1,2,4,5,7,8,9]. Current Surviving Sepsis Campaign guidelines recommend against routine use of iNO in ARDS due to lack of demonstrated survival benefit. However, it may be considered as rescue therapy in refractory hypoxemia [10]. While generally well tolerated, iNO has been associated with adverse outcomes, including acute kidney injury, hospital-acquired pneumonia, and potential rebound hypoxemia with abrupt discontinuation [1,2,8,10].

In addition to its use in mechanically ventilated patients, iNO has also been evaluated in spontaneously breathing, non-intubated patients with COVID-19-associated hypoxemia, with some studies suggesting a potential role in improving oxygenation and reducing escalation to invasive mechanical ventilation in select patients [2]. Although the present study focuses on intubated patients, this body of literature underscores the broader relevance of iNO across the spectrum of COVID-19 respiratory failure severity.

The use of iNO in ARDS predates the COVID-19 pandemic. Previous randomized controlled trials have established iNO as an effective way of improving oxygenation without systemic vasodilation. However, landmark trials, including those by Lundin et al. and Taylor et al., failed to demonstrate a mortality benefit [11,12]. Additionally, a Cochrane meta-analysis similarly concluded that iNO does not reduce mortality and may increase the risk of renal dysfunction [9]. Despite this, iNO has remained in clinical use as a rescue therapy for refractory hypoxemia, particularly as a bridge to ECMO or as a temporizing measure during prone positioning. The emergence of COVID-19-associated ARDS renewed interest in iNO given the prominent role of hypoxic pulmonary vasoconstriction and ventilation–perfusion mismatch in its pathophysiology.

COVID-19-associated ARDS is characterized by diffuse alveolar damage, microvascular thrombosis, and dysregulated inflammation, resulting in severe ventilation–perfusion mismatch and refractory hypoxemia [5]. Pulmonary vascular dysfunction, including endothelial injury and in situ thrombosis, contributes to elevated pulmonary vascular resistance and right ventricular strain in a subset of patients [7]. Inhaled nitric oxide selectively vasodilates pulmonary vessels in ventilated lung units, redistributing perfusion away from non-ventilated regions and, as a result, improving the PaO_2_/FiO_2_ ratio without systemic hemodynamic effects. These physiologic properties make iNO a rational rescue therapy in COVID-19-associated ARDS, particularly in patients with evidence of pulmonary hypertension or right ventricular dysfunction.

Most available data are derived from retrospective and observational studies, with relatively few randomized trials. Additionally, significant heterogeneity in patient selection, dosing protocols, and initiation timing limits the interpretation of existing evidence [13]. However, the impact of iNO initiation timing on clinical outcomes remains poorly defined, particularly in patients with COVID-19 who require mechanical ventilation.

While prior studies have primarily examined whether iNO improves oxygenation or survival, comparatively little attention has been given to whether the timing of iNO initiation relative to intubation independently influences clinical outcomes. This represents a gap in the existing literature, as timing may modify the therapeutic window during which pulmonary vasodilation can meaningfully improve ventilation–perfusion matching before disease progression becomes refractory to intervention.

This retrospective cohort study expands on existing work by evaluating both patient characteristics associated with mortality and the timing of iNO initiation as potential determinants of clinical outcomes. This study aimed to evaluate the association between the timing of inhaled nitric oxide initiation and clinical outcomes in mechanically ventilated patients with COVID-19.

## 2. Materials and Methods

### 2.1. Study Design and Setting

A retrospective cohort study was conducted of adult patients admitted to the medical intensive care unit (MICU) at University Medical Center in Lubbock, TX, with confirmed COVID-19 who received inhaled nitric oxide (iNO) during invasive mechanical ventilation from 1 January 2020 through 31 December 2022. The study was approved by the Texas Tech University Health Sciences Center Institutional Review Board (L23-096; approval date 20 March 2023) with a waiver of informed consent due to minimal risk.

### 2.2. Patient Selection

Patients were eligible for inclusion if they met all of the following criteria:

- Age 18 years of age or older;
- Confirmed SARS-CoV-2 infection by polymerase chain reaction (PCR);
- Required invasive mechanical ventilation;
- Received iNO at any time during their ICU course.

Of 101 patients initially identified, patients were excluded if either of the following occurred:

- They did not have confirmed COVID-19 (*n* = 3);
- If outcome data were unavailable (*n* = 4).

This yielded a final analytic cohort of 94 patients.

ARDS was defined according to the Berlin criteria, requiring an acute onset within 1 week of a known clinical insult, bilateral opacities on chest imaging not fully explained by effusions or lobar collapse, and a PaO_2_/FiO_2_ ratio less than 300 mmHg with a minimum of 5 cmH_2_O positive end-expiratory pressure (PEEP). Severity was classified as mild (PaO_2_/FiO_2_ 200–300), moderate (PaO_2_/FiO_2_ 100–200), or severe (PaO_2_/FiO_2_ less than 100) [14].

### 2.3. Data Collection and Variables

Clinical data were abstracted from the electronic medical record and recorded in a standardized database. Variables collected included demographic characteristics (age, sex, race, body mass index), comorbidities (including hypertension, diabetes mellitus, chronic obstructive pulmonary disease (COPD), obstructive sleep apnea (OSA), and cardiac function assessed by left ventricular ejection fraction (EF)), and severity of illness scores (Sequential Organ Failure Assessment (SOFA) and Acute Physiology and Chronic Health Evaluation II (APACHE II)).

COPD status was determined based on documented past medical history. Pulmonary function tests were reviewed when available to corroborate the diagnosis but were available for only 3 patients; diagnosis for the remainder of the cohort therefore relied on chart documentation, which is noted as a limitation. Left ventricular ejection fraction was assessed via formal transthoracic echocardiography performed at the bedside by an echocardiography technologist and interpreted by a cardiologist using Simpson’s biplane method; echocardiograms were available for 82 of 94 patients (87.2%).

Treatment-related variables included mechanical ventilation duration, hospital length of stay (LOS), iNO therapy duration, and timing of iNO initiation. Timing of iNO initiation was defined as the number of days from endotracheal intubation to iNO initiation.

Inhaled nitric oxide was administered via a dedicated delivery system integrated into the mechanical ventilator circuit. Per institutional protocol, iNO was initiated at a starting dose of 20 parts per million (ppm) in patients with refractory hypoxemia, defined as a persistent PaO_2_/FiO_2_ ratio below 150 mmHg despite optimization of PEEP, FiO_2_, and ventilator settings. Dose titration was performed at the treating intensivist’s discretion based on the oxygenation response, with a maximum dose of 40 ppm. Weaning of iNO was performed gradually to avoid rebound hypoxemia with dose reductions at intervals determined by clinical response. Discontinuation was considered when the PaO_2_/FiO_2_ ratio remained above 200 mmHg on reduced iNO doses, or care was transitioned to comfort-focused. The P/F ratio threshold of 150 mmHg was applied as a general institutional guideline; individual clinicians retained discretion over the timing of iNO initiation based on the overall clinical context.

Adjunctive therapies administered during the ICU course were captured from the medical record and included prone positioning, neuromuscular blockade, systemic corticosteroids, and antiviral therapy. Lung-protective ventilation strategies were employed per institutional protocol, targeting tidal volumes of 6 mL/kg of ideal body weight and plateau pressures below 30 cmH_2_O in accordance with ARDS Network guidelines [15]. The use of these therapies was documented but was not used as an exclusion criterion, as the goal of this study was to evaluate outcomes in an everyday clinical population.

### 2.4. Outcomes

The primary outcome was in-hospital mortality. Secondary outcomes included mechanical ventilation duration, hospital length of stay, and iNO therapy duration. Additional analyses evaluated the association between the timing of iNO initiation and survival, as well as the relationship between patient characteristics and mortality.

### 2.5. Statistical Analysis

Continuous variables are summarized as mean and standard deviation or median and interquartile range (IQR), as appropriate. Categorical variables are presented as counts and percentages and compared using chi-square or Fisher’s exact tests, as appropriate.

Survival analyses were performed using Kaplan–Meier curves, with differences between groups evaluated using the log-rank test. The time-to-event outcome was defined as the number of days from ICU admission to in-hospital death. Patients discharged alive were censored at the date of hospital discharge. Cox proportional hazards regression models were used to estimate associations between clinical characteristics and time to in-hospital death, with results reported as hazard ratios (HRs) and 95% confidence intervals (CIs). Multivariable models were adjusted for relevant demographic and clinical covariates, including age, sex, BMI, COPD, OSA, hypertension, diabetes, and ejection fraction.

A two-sided *p*-value < 0.05 was considered statistically significant. Multiple testing adjustments were not performed. All statistical analyses were performed using standard statistical software [16].

### 2.6. AI Usage Declaration

Generative AI tools, including OpenEvidence (Osler) and Claude (Sonnet 5, Anthropic), were used to assist with literature review and during manuscript preparations to assist with grammar and language editing, structural formatting review for journal compliance, and reference formatting guidance. All scientific content, data analysis, interpretations, and conclusions were generated solely by the authors.

## 3. Results

### 3.1. Patient Characteristics

A total of 94 mechanically ventilated patients with confirmed COVID-19 who received iNO were included in the analysis. The mean age was 52.0 years (SD 14.1), and the cohort was predominantly male (70.2%) and obese, with a mean body mass index (BMI) of 36.5 kg/m^2^ (SD 10.0). The majority of patients were identified as Hispanic (51.1%), followed by White (30.9%) and Black (7.4%).

Hypertension (58.5%) and diabetes mellitus (36.2%) were the most common comorbidities, while COPD (4.3%) and OSA (6.4%) were less prevalent. Most patients had preserved left ventricular ejection fraction (>50%) (78.7%), with reduced ejection fraction (≤50%) observed in 4.3% of patients. Ejection fraction was unknown in 17% of patients. Baseline characteristics are summarized in Table 1.

Chest radiographs obtained at the time of ICU admission demonstrated bilateral infiltrates in 86 of 94 patients (91.5%), with bilateral alveolar infiltrates in 81.9% of the cohort, consistent with severe ARDS. A small number of patients had additional radiographic findings, including pneumothorax (2.1%) and pleural effusion (2.1%).

### 3.2. Clinical Outcomes

In-hospital mortality was 85.1% (80 of 94), with 14 patients surviving to hospital discharge.

### 3.3. Predictors of In-Hospital Mortality

Baseline demographic characteristics, including age, sex, BMI, and race, were not significantly associated with mortality. Similarly, hypertension, diabetes mellitus, and OSA were not significantly associated with survival. However, COPD was associated with a significantly increased risk of mortality (hazard ratio (HR) 4.20, 95% confidence interval (CI) 1.14–15.47; *p* = 0.031). In contrast, preserved ejection fraction (>50%) was strongly associated with improved survival (HR 0.09, 95% CI 0.03–0.30; *p* < 0.001). These findings are summarized in Table 2.

### 3.4. Kaplan–Meier Survival Analysis

**Figure 1:**
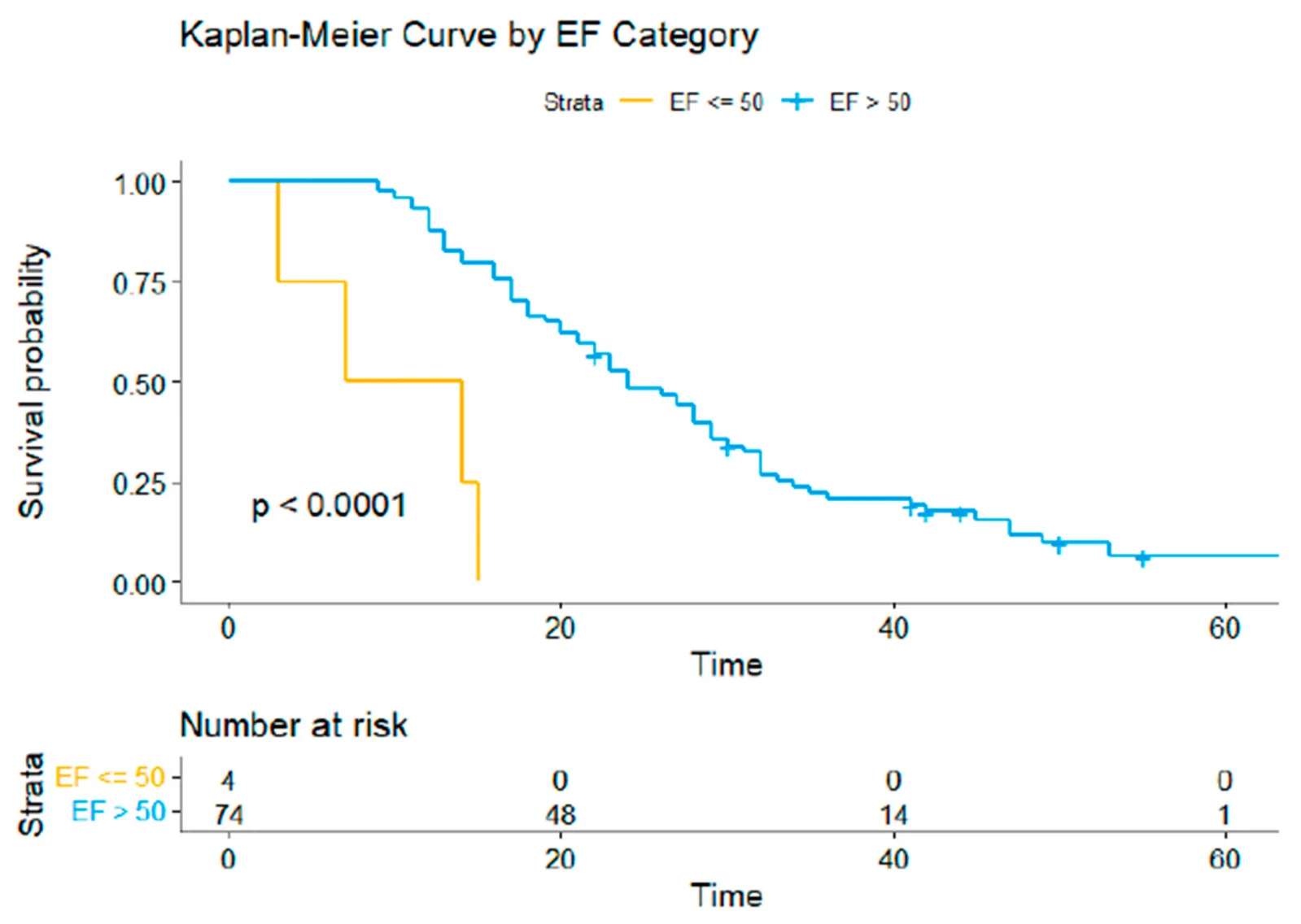
Kaplan–Meier Curve by EF.

Kaplan–Meier survival analysis demonstrated that patients with reduced ejection fraction (≤50%) had markedly worse survival compared to those with preserved ejection fraction (>50%) (log-rank *p* < 0.0001). Survival in the reduced ejection fraction group declined rapidly, with no patients surviving beyond approximately 20 days, whereas patients with preserved ejection fraction demonstrated improved survival over time. Data are shown in Figure 1.

**Figure 2:**
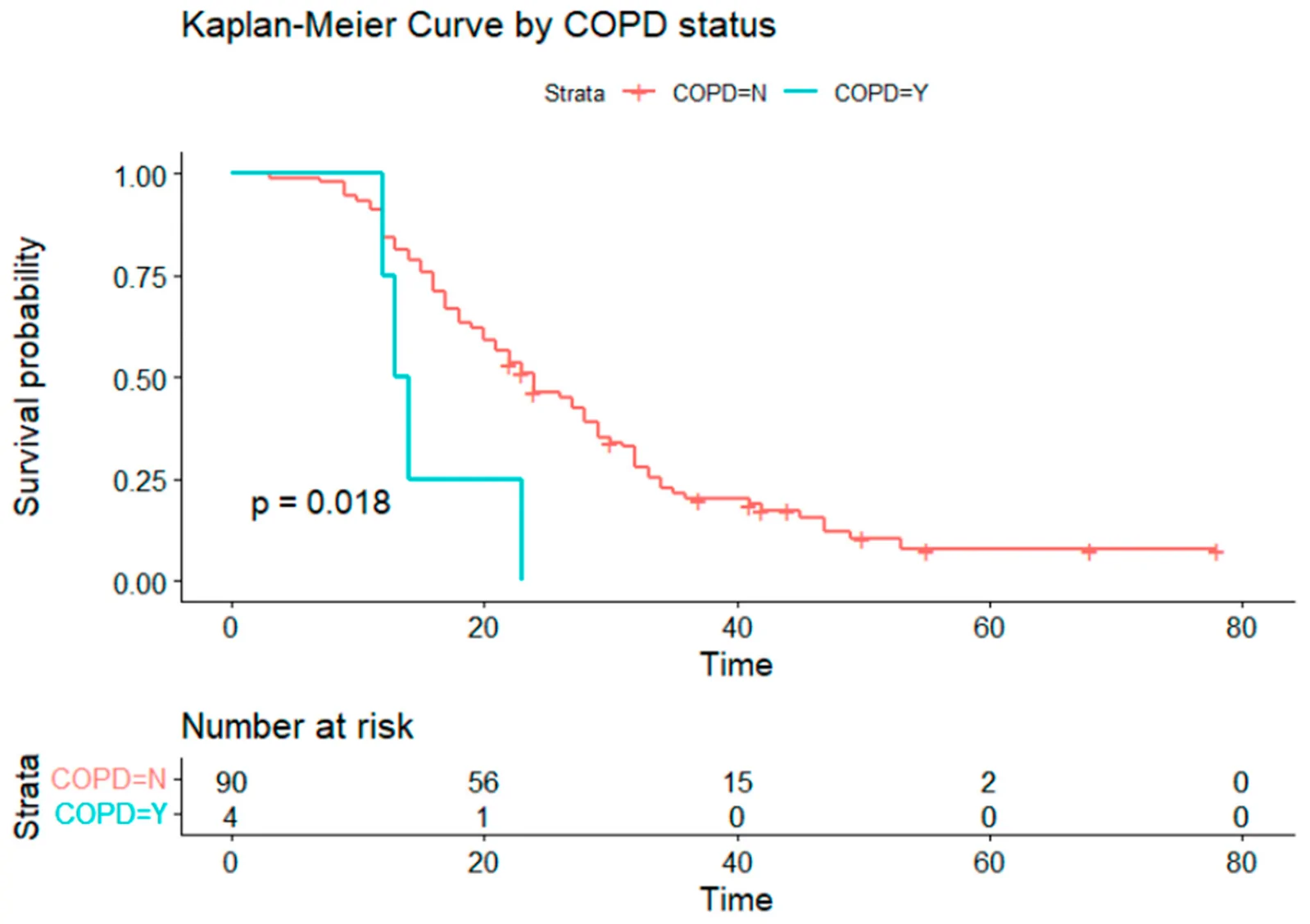
Kaplan–Meier Curve by COPD.

Similarly, Kaplan–Meier survival analysis demonstrated significantly reduced survival among patients with COPD compared to those without COPD (log-rank *p* = 0.018). All patients with COPD died within approximately 25 days of ICU admission. Data are shown in Figure 2.

### 3.5. Timing of iNO Initiation and Outcomes

The association between the timing of iNO initiation and clinical outcomes was next evaluated. The median time from intubation to initiation of iNO was 7.6 days (mean 7.7 days, SD 9.2). Survivors received iNO significantly earlier than non-survivors (median 0.5 vs. 7 days; mean 4.14 vs. 8.3 days after intubation, *p* = 0.024). Duration of iNO therapy did not differ significantly between survivors and non-survivors (*p* = 0.12).

Serial arterial blood gas data were available for a subset of patients (*n* = 52 at ICU admission, *n* = 56 pre-iNO, *n* = 56 post-iNO). Patients with missing ABG data were not systematically different from those with available data in age, sex, or mortality rate, though formal comparisons were limited by sample size. At the time of ICU admission, the mean PaO_2_/FiO_2_ ratio was 75.3 mmHg (SD 49.8; median 61.0), with 82.7% of patients meeting criteria for severe ARDS (PaO_2_/FiO_2_ less than 100 mmHg) and 15.4% meeting criteria for moderate ARDS (PaO_2_/FiO_2_ 100–200 mmHg). Immediately prior to iNO initiation, the mean PaO_2_/FiO_2_ ratio was 64.0 mmHg (SD 26.3; median 57.5), reflecting persistent severe hypoxemia despite mechanical ventilation and other supportive measures. Among the 56 patients with paired pre- and post-iNO arterial blood gas data, 38 patients (67.9%) demonstrated an oxygenation response, defined as a >20% improvement in the PaO_2_/FiO_2_ ratio within 24–48 h of iNO initiation. The mean PaO_2_/FiO_2_ ratio at 24–48 h post-iNO initiation was 95.5 mmHg (SD 42.2; median 85.0), representing a mean increase of 31.5 mmHg from pre-iNO values.

### 3.6. Secondary Outcome

Survivors had a significantly longer hospital length of stay compared to non-survivors (mean 37 vs. 23 days, *p* < 0.01), consistent with the earlier iNO initiation observed among survivors noted above.

## 4. Discussion

This retrospective cohort study of mechanically ventilated patients with COVID-19 who received iNO observed an extremely high in-hospital mortality rate of 85.1%. Earlier initiation of iNO after intubation was associated with improved survival, whereas the duration of therapy was not. In addition, patient-specific factors, including COPD and left ventricular ejection fraction, were strongly associated with mortality.

All patients had a confirmed SARS-CoV-2 infection by PCR and a clinical ARDS diagnosis at the treating team’s discretion, but alternative or contributing etiologies of ARDS were not systematically excluded, which may introduce diagnostic heterogeneity. Additionally, this study did not include a comparator group of ARDS patients who did not receive iNO and therefore could not directly assess whether iNO confers a mortality benefit relative to no treatment. However, the absence of an association between iNO therapy duration and survival within this cohort of iNO recipients is consistent with prior literature describing transient oxygenation improvements with iNO that have not translated into reduced mortality or shorter mechanical ventilation duration.

However, this study expands upon existing literature by examining the timing of iNO initiation as a potential determinant of clinical outcomes. Patients who received iNO earlier in their mechanical ventilation course had better survival than those who received it later. One possible explanation is that earlier administration of iNO may improve ventilation–perfusion matching and reduce hypoxic pulmonary vasoconstriction at a stage when lung injury is potentially more reversible. In contrast, later initiation of iNO may reflect more advanced or refractory disease, in which pulmonary vascular dysregulation and parenchymal injury are less responsive to targeted vasodilation. Alternatively, these associations are subject to important confounding, including a form of immortal time bias. Patients who died within the first few days of mechanical ventilation never had the opportunity to receive late iNO; their deaths would be categorized alongside early iNO initiation by default, potentially inflating the apparent survival benefit of early timing. A formal landmark analysis starting at a fixed timepoint post-intubation (such as day 3 or day 5) would be required to address this rigorously and was not performed in the current study. Additionally, patients receiving iNO later in their disease course may represent a population with more refractory illness and a worse underlying prognosis, independently of iNO timing. Conversely, earlier initiation may reflect differences in clinician practice patterns or institutional protocols, or earlier recognition of hypoxemia rather than a direct therapeutic effect. Therefore, the observed association between earlier iNO initiation and improved survival should be considered hypothesis-generating rather than causal.

This timing finding has not been a primary focus of prior studies and represents a meaningful addition to the existing literature. Mekontso Dessap et al. evaluated iNO in COVID-19 ARDS across multiple centers and reported that oxygenation response did not predict survival, but did not examine the timing of initiation as an independent variable [5]. Similarly, Al Sulaiman et al. reported no mortality benefit from iNO in a multicenter cohort but noted significant heterogeneity in the timing of iNO initiation relative to intubation [1]. Di Fenza et al., in a phase II trial of high-dose iNO, demonstrated improved oxygenation in a subset of patients but did not identify a survival benefit, and timing of initiation was not systematically analyzed [9]. Taken together, these studies establish that iNO improves oxygenation but not mortality; the current findings suggest that the timing of initiation may be an insufficiently evaluated variable that could explain some of the heterogeneity in outcomes across studies.

COPD was also identified as a significant risk factor for mortality and preserved ejection fraction as strongly protective. These findings emphasize the importance of underlying cardiopulmonary comorbidities in determining outcomes in critically ill patients with COVID-19. Patients with COPD may have limited pulmonary reserve and impaired gas exchange, which could exacerbate the severity of hypoxemic respiratory failure. In contrast, preserved cardiac function may support better hemodynamic stability and oxygen delivery in the setting of severe illness. However, these findings should be interpreted with caution. Only 4 patients in our cohort had COPD, and none survived, resulting in a wide confidence interval for the hazard ratio (HR 4.20, 95% CI 1.14–15.47). The association is statistically significant, but clinically meaningful conclusions are limited by the small number of events; a single additional COPD survivor would substantially alter this estimate. Similarly, the EF analysis is constrained by the small number of patients with reduced EF (*n* = 4) and the 17% of patients with unknown EF who were excluded from this analysis. Further studies with larger cohorts are needed to confirm whether COPD and reduced EF independently predict mortality in this population.

These findings have potential clinical implications for the use of iNO in critically ill patients with COVID-19 ARDS. If earlier initiation is associated with improved outcomes, clinicians may consider earlier deployment of iNO in selected patients rather than reserving it exclusively for refractory or late-stage hypoxemia. This would represent a shift from the current practice of using iNO solely as a rescue therapy after other interventions have failed. However, given the retrospective nature of this study and the potential for confounding, this hypothesis requires prospective validation before recommending changes to clinical practice. Identifying the optimal threshold for initiation, whether defined by P/F ratio, duration of mechanical ventilation, or failure to respond to prone positioning, will be an important focus of future research.

The mean PaO_2_/FiO_2_ ratio immediately prior to iNO initiation was 64.0 mmHg, confirming that iNO was administered in the setting of severe refractory hypoxemia consistent with our institutional threshold. The oxygenation response rate of 67.9% in our cohort modestly exceeds the 45–66% range reported in prior studies of iNO in COVID-19 ARDS [1,2,3,4,5,6,7], which may reflect differences in patient selection, ARDS severity, or the timepoint at which oxygenation response was assessed. This finding nevertheless supports the generalizability of these findings and suggests that the patient population responded to iNO in a manner broadly comparable to previously described cohorts. Notably, despite this oxygenation response, overall mortality remained extremely high, further reinforcing that improvements in the PaO_2_/FiO_2_ ratio do not reliably translate into a survival benefit, a finding consistent with the broader literature.

Prospective studies are needed to evaluate whether earlier, protocolized initiation of iNO improves survival compared to current standard practice. Future studies should incorporate standardized ARDS definitions, consistent dosing protocols, and systematic documentation of initiation timing to allow for meaningful comparisons across centers. Biomarker-guided approaches, such as identifying patients with elevated pulmonary vascular resistance or echocardiographic evidence of right ventricular dysfunction, may help define the subset of patients most likely to benefit from iNO, particularly those with early intubation and refractory hypoxemia. Additionally, the relationship between iNO and adjunctive therapies, such as prone positioning, neuromuscular blockade, and corticosteroids, warrants further investigation, as these interventions may interact with the timing and efficacy of iNO.

It is also worth noting that inhaled nitric oxide has toxic effects at high doses or in specific contexts. Nader et al. demonstrated that high-dose nitric oxide at 80 ppm increases lung injury and protein permeability in an animal model after gastric aspiration, with additional injury from concurrent hyperoxia [17]. This dose is twice the maximum used in this cohort (40 ppm), and this study involved COVID-19-associated ARDS rather than aspiration injury; therefore, direct extrapolation is limited. Nevertheless, this finding demonstrates the importance of dose optimization and careful patient selection when administering iNO, particularly in patients with concurrent hyperoxia or additional mechanisms of lung injury [17].

### 4.1. Study Limitations

This study has several important limitations. First, its retrospective, single-center design limits generalizability and introduces the potential for selection bias. Second, the absence of a control group of patients who did not receive iNO precludes assessing the overall effectiveness of iNO compared with standard care. Third, the timing analysis is subject to immortal time bias: patients who died early could not have received late iNO initiation, which may have confounded the association between timing and survival. A landmark analysis was not performed and should be prioritized in future work. Fourth, unmeasured confounders, including hypoxemia severity at iNO initiation, ventilator management strategies, and adjunctive therapies, may have influenced outcomes. Fifth, serial ABG data were available for only a subset of patients, limiting the generalizability of the oxygenation response analyses. Additionally, the small sample size, particularly within subgroups such as patients with COPD (*n* = 4) or reduced ejection fraction (*n* = 4), limits statistical power and results in unstable hazard ratio estimates with substantial uncertainty. The observed associations for COPD and ejection fraction should therefore be interpreted cautiously, as a small change in the number of events could alter the estimated effect sizes. These findings should be considered exploratory and hypothesis-generating rather than definitive evidence of independent prognostic effects.

Additionally, this study did not capture the overall in-hospital mortality rate among all ARDS patients admitted to the participating ICU during the study period, so it is not possible to determine whether mortality among iNO recipients differed from unit-wide ARDS mortality. Code status was not systematically captured; given that refractory hypoxemia often prompts goals-of-care discussions, this limits interpretation of the relationship between disease trajectory and end-of-life decision-making within this cohort. Echocardiographic assessment of right ventricular function and pulmonary artery pressures was not consistently available across the cohort and could not be incorporated into this analysis; baseline pulmonary hypertension may have influenced the observed association between iNO timing and survival.

### 4.2. Recommendations for Further Research

Despite these limitations, this study provides clinical information regarding the potential role of timing in iNO administration. Earlier initiation of iNO was associated with improved survival, whereas duration of therapy was not, suggesting that timing, rather than prolonged use, may be a more important determinant of response. Given that iNO is frequently used as a rescue therapy, these findings suggest that earlier use in selected patients may improve outcomes. Prospective studies are needed to evaluate the impact of timing further and to determine whether earlier initiation of iNO can lead to meaningful clinical benefit. Future analyses should also consider incorporating early versus late iNO initiation as a covariate within the multivariable Cox model to better isolate its independent association with survival.

## 5. Conclusions

In this cohort of critically ill patients with COVID-19 and severe respiratory failure, overall mortality was extremely high. COPD was associated with increased mortality, and preserved ejection fraction on transthoracic echocardiogram was associated with decreased mortality. Earlier initiation of inhaled nitric oxide was associated with improved survival, whereas duration of therapy was not. These findings suggest that the timing of iNO administration may be associated with clinical outcomes and warrant further prospective investigation to confirm this association.

## Figures and Tables

**Table 1:** Patient Characteristics.

Variable	Value
Age, mean (SD), years	52.0 (14.1)
BMI, mean (SD), kg/m^2^	36.5 (10.0)
Age > 55, *n* (%)	42 (44.7%)
BMI category, *n* (%)	
Normal (18.5–24.9)	8 (8.5%)
Overweight (25–29.9)	21 (22.3%)
Obese (≥30)	62 (66.0%)
Unknown	3 (3.2%)
Sex, *n* (%)	
Female	28 (29.8%)
Male	66 (70.2%)
Race, *n* (%)	
Black	7 (7.4%)
Hispanic	48 (51.1%)
White	29 (30.9%)
Other/unknown	10 (10.6%)
COPD, *n* (%)	4 (4.3%)
OSA, *n* (%)	6 (6.4%)
Hypertension, *n* (%)	55 (58.5%)
Diabetes mellitus, *n* (%)	34 (36.2%)
Ejection fraction, *n* (%)	
≤50%	4 (4.3%)
>50%	74 (78.7%)
Unknown	16 (17.0%)

**Table 2:** MICU mortality risk factors.

Variable	Alive (*n* = 14)	Expired (*n* = 80)	Adjusted HR (95% CI)	*p* Value
Age	50.0 (13.3)	52.3 (14.3)	1.00 (0.98, 1.03)	0.81
BMI: Normal	1 (7.1%)	7 (8.8%)	Reference	
BMI: Overweight	3 (21.4%)	18 (22.5%)	1.15 (0.44, 3.0)	0.771
BMI: Obese	10 (71.4%)	52 (65.0%)	0.83 (0.32, 2.18)	0.708
Sex: Female	4 (28.6%)	24 (30.0%)	Reference	
Sex: Male	10 (71.4%)	56 (70.0%)	1.04 (0.59, 1.83)	0.893
Race: Black	2 (14.3%)	5 (6.2%)	Reference	
Race: Hispanic	9 (64.3%)	39 (48.8%)	1.00 (0.38, 2.63)	0.995
Race: White	1 (7.1%)	28 (35.0%)	1.63 (0.60, 4.44)	0.34
COPD: No	14 (100%)	76 (95.0%)	Reference	
COPD: Yes	0 (0.0%)	4 (5.0%)	4.20 (1.14, 15.47)	0.031
OSA: No	13 (92.9%)	75 (93.8%)	Reference	
OSA: Yes	1 (7.1%)	5 (6.2%)	1.29 (0.45, 3.71)	0.642
HTN: No	4 (28.6%)	35 (43.8%)	Reference	
HTN: Yes	10 (71.4%)	45 (56.2%)	0.94 (0.53, 1.7)	0.849
DM: No	8 (57.1%)	52 (65.0%)	Reference	
DM: Yes	6 (42.9%)	28 (35.0%)	1.33 (0.77, 2.3)	0.311
EF: ≤50	0 (0.0%)	4 (5.0%)	Reference	
EF: >50	10 (71.4%)	64 (80.0%)	0.09 (0.03, 0.3)	<0.001

## Data Availability

Data are available upon request.
